# Effectiveness of survivorship programmes to enhance health-related quality of life of colorectal cancer survivors: a systematic review and meta-analysis of randomised controlled trials

**DOI:** 10.1007/s00520-026-10779-8

**Published:** 2026-05-18

**Authors:** Pham Nhat Vi Do, Thi Khanh Nguyen, Yishu Qi, Kai Chow Choi, Thi Xuan Huong Hoang, Ba Phuoc Le, Yuen Tung Lam, Mankei Tse, Cho Lee Wong

**Affiliations:** 1 The Nethersole School of Nursing, Faculty of Medicine, The Chinese University of Hong Kong, Hong Kong SAR, China; 2 Phenikaa University, Hanoi, Vietnam; 3 The Ho Chi Minh Oncology Hospital, No Trang Long Street, Binh Thanh District, Ho Chi Minh City, Vietnam

**Keywords:** Survivorship, Colorectal cancer, Survivors, Health-related quality of life, Systematic review, Meta-analysis

## Abstract

**Purpose:**

This study aimed to assess the effectiveness of the survivorship programmes by synthesising and analysing the available evidence to enhance health-related quality of life (HRQoL) amongst colorectal cancer (CRC) survivors.

**Methods:**

Ten English-language databases were searched from inception to June 2025 in this systematic review and meta-analysis. All randomised controlled trials (RCTs) were included. Available data were pooled in a meta-analysis using RevMan (version 5.4.0). Two independent reviewers performed the database searches, extracted the data, assessed the methodological quality by using the Cochrane Risk-of-Bias tool (version 2) and evaluated the overall quality of findings by using Cochrane GRADE.

**Results:**

A total of 22 RCTs involving 2949 CRC survivors were identified. The meta-analysis results (thirteen studies) indicated a significant improvement in the physical (standardised mean difference [SMD] = 0.52, 95% confidence interval [CI, 0.18, 0.86], *P* = 0.002, *I*^2^ = 88%) and mental domains of HRQoL (SMD = 0.4, 95% CI [0.06, 0.74], *P* = 0.02, *I*^2^ = 86%). Sensitivity analysis involved reducing the heterogeneity after removing one study. No publication bias was found. The overall quality of findings was from ‘low’ to ‘moderate’.

**Conclusions:**

The detailed components of the survivorship programme can enhance HRQoL in physical and mental domains, indicating its potential as valuable evidence for health providers to support CRC survivors post-treatment. Future research should focus on expanding the delivery of such programme comprehensively by integrating mobile health into a nurse-led approach to optimise geographic diversity and improve social HRQoL.

**Supplementary information:**

The online version contains supplementary material available at 10.1007/s00520-026-10779-8.

## Introduction

Colorectal cancer (CRC) is the third leading cause of cancer-related deaths for both men and women [1]. By 2030, the global burden of CRC is expected to increase by 60%, leading to an additional 2.2 million new cases and 1.1 million deaths [2]. Despite these alarming statistics, advancements in CRC treatments, including laparoscopic surgery, radiation therapy and chemotherapy, have remarkably improved the long-term survival rates for CRC survivors, marking what is known as cancer survivorship [3]. The overall 5-year survival rate ranges from 40 to 70% in countries [4, 5].

Cancer survivorship has many definitions. Mullan introduced the first term, outlining three phases: the acute phase, the extended phase and permanent survival [6]. In 2006, the Institute of Medicine expanded this definition to encompass the entire journey from initial diagnosis to the end of life [7]. However, most studies define the survivorship phase as the time following the completion of primary treatment (surgery, chemotherapy and radiotherapy) [8–11]. This review adopts the widely accepted survivorship definition, focusing on survivors who have completed primary treatment. The post-primary treatment period can be particularly challenging for those with a CRC diagnosis, reducing their health-related quality of life (HRQoL) [12]. HRQoL encompasses an individual’s perception of their life across physical, mental and social dimensions, aligning with the criteria outlined in WHO’s definition of health [13–15]. Several factors contribute to this decrease, including unmet information needs, limited access to support from healthcare providers after treatment [16] and the experience of CRC survivors’ symptoms, such as depression, anxiety, distress, fatigue and bowel dysfunction [8, 17].

With the growing population of CRC survivors, providing effective programmes to address their needs is essential. The Institute of Medicine recommends the development of survivorship programmes specifically to meet the supportive care needs of this group [18]. Since then, various organisations, including the National Comprehensive Cancer Network and the American Society of Clinical Oncology, have released guidelines to address these critical topics. Building upon previous recommendations, the American Cancer Society (ACS) released its CRC Survivorship Care Guidelines, recognising the gaps in survivorship resources and clinical follow-up in post-treatment. This guideline provides comprehensive care recommendations to help CRC survivors achieve optimal health and HRQoL. The key dimensions focus on four main areas: (1) surveillance for CRC recurrence and screening for second cancers, (2) management of the long-term physical and psychological effects of CRC treatment, (3) promotion of healthy behaviours and (4) coordination of care between specialists and primary care clinicians [19].

After the ACS guidelines were instituted in 2015, the systematic reviews of survivorship programmes have focused on only a single aspect of it, such as managing late effects or promotion of health, rather than the four dimensions [20–22]. As a result, survivors’ supportive needs are often not comprehensively addressed, which can lead to inappropriate treatment decisions or reduced treatment effectiveness, ultimately affecting their adherence and HRQoL [23]. The umbrella term ‘colorectal cancer patients’ in current studies includes those who are undergoing treatment or adjuvant chemotherapy/radiotherapy and those who have completed treatment [24, 25]. However, the needs of the two groups are different. Whilst the former group requires a focus on targeted therapy and management of side effects during treatment, the latter group requires more as they return to a ‘new normal’ life [26, 27]. Therefore, focusing on each group individually is necessary to meet their unmet needs.

Meanwhile, the implementation in research and practice following the ACS guidelines remains limited. To the authors’ knowledge, a prior literature review provides an overview of the survivorship programme for CRC survivors post-treatment on the basis of ACS’s CRC survivorship care guidelines, which synthesised data from five databases, including 30 studies (five reviews, 12 randomised controlled trials [RCTs] and 13 non-RCTs). The results, as for the long-term physical and psychosocial effects of CRC treatment, indicated that the programmes could address ostomy issues and improve the distress, depression and anxiety of survivors. For health promotion, exercise can effectively reduce fatigue and improve HRQoL in the short term of less than three months [28]. The CRC surveillance programmes needed to be explored, and the care coordination programme showed improvement through survivorship care plan. However, that review was constrained by heterogeneous study designs (systematic reviews, RCTs and non-RCTs) and a limited search strategy (five databases), giving uncertain conclusions. Crucially, neither the ACS guidelines nor the existing literature review provides detailed guidance on key intervention components such as dosage, format, facilitators or delivery mode. This lack of detail leaves healthcare providers and survivors with inadequate support, which ultimately affects long-term HRQoL.

To address this gap, this systematic review aimed to synthesise the available evidence on the effects of survivorship programmes, compared with usual care, on health-related quality of life among colorectal cancer survivors.

## Methods

This systematic review followed the guidelines for reporting parallel group randomised trials on the basis of the Preferred Reporting Items for Systematic Reviews and Meta-Analyses (PRISMA) statement [29]. A prospective protocol was registered with the International Prospective Register of Systematic Reviews (PROSPERO ID: CRD42024580272).

### Inclusion and exclusion criteria

This review included RCTs that focused on survivorship programmes aimed at managing HRQoL amongst CRC survivors. The exclusion criteria included conference abstracts, protocols, surveys, review studies and studies that lacked pre- and post-intervention assessments or did not report HRQoL. The details of the inclusion and exclusion criteria are described in Supplementary File 1.

### Data sources

A systematic search was performed across 10 databases (PubMed, Cochrane Library, Scopus, CINALH Ultimate, Embase, OVID EMCARE, OVID Nursing, OVID Medline, PsycINFO and Web of Science) by using specific search strategies from the databases’ inception to June 2025. The reference lists of identified articles were manually searched by citation of the included studies’ reference lists and conducted forward citations to find any additional relevant studies not retrieved by the electronic database searches. The search utilised titles, abstracts, keywords, MeSH terms and no-date restrictions.

### Search strategies

The search keywords for the databases included the following: (‘Colorectal cancer survivor’ OR ‘cancer’ OR ‘neoplasm’ OR ‘colon’ OR ‘rectal’ OR ‘colorectal’ OR ‘Cancer Survivors[Mesh]’) AND (‘survivorship’ OR ‘post-treatment’ OR ‘follow-up’ OR ‘Survivorship[Mesh]’ OR ‘intervention’) AND (‘RCT’ OR ‘Randomized Controlled Trial’) AND (‘health-related quality of life’ OR ‘Quality of life’). Further details about the search strategy are presented in Supplementary File 2.

### Study selection

The PRISMA guidelines for reporting parallel group randomised trials were followed. After the database searches were completed, duplicate studies were removed manually using EndNote 20 software. Two independent reviewers (D.P.N.V. and K.N.T.) screened the remaining articles on the basis of the title and abstract related to the inclusion criteria. The full texts of potentially eligible articles were then downloaded and assessed for inclusion. Whenever the two reviewers could not reach an agreement, a third reviewer (W.C.L.) was consulted to make a final decision by majority.

### Data extraction

Data extraction and summarisation were carried out independently by the two reviewers (D.P.N.V. and K.N.T.). If a consensus could not be reached, the opinion of the third reviewer (W.C.L.) was obtained. The extracted data included participant demographics; disease status; and details of the intervention, including components, duration, providers, formats and outcome measures.

### Methodological quality

The Risk-of-Bias (version 2.0) tool was used to assess the methodological quality, focusing on trial design, conduct and reporting. The questions within each domain gathered information relevant to the risk of bias. On the basis of the responses, the tool generated proposed judgments of ‘low risk’, ‘high risk’ or ‘some concerns’ for each domain. Disagreements between the two reviewers were resolved through discussions with the third reviewer. The Grading of Recommendation, Assessment, Development and Evaluation (GRADE) system was used to assess the quality of evidence, which was classified with four levels: high, moderate, low and very low. The following five factors subsequently downgraded the initially high-quality findings from RCTs: risk of bias, inconsistency, indirectness, imprecision and biased reporting [30].

### Data analysis

RevMan (version 5.4) was used to pool the quantitative results for statistical meta-analysis. Standardised mean differences (SMDs) and 95% confidence intervals (CIs) were calculated because the studies reported continuous data with non-identical outcome measures. For insufficient data for pooling meta-analysis, the original authors of the included studies were contacted. Clinical heterogeneity was assessed on the basis of similarities in participants, settings, interventions and outcomes. Statistical heterogeneity was assessed via *I*^2^, which revealed percentages of 25%, 50% and 75% as low, moderate and high levels of heterogeneity, respectively [31]. Given the variability of survivorship programmes across the studies, data were pooled using a random-effect model [32]. SMDs of values 0.2, 0.5 and 0.8 were considered small, medium and large effects, respectively [33]. Data were pooled from the subscales of HRQOL instrument and categorised into the physical, psychological/emotional and social domains in meta-analysis. Subgroup analyses were conducted for the components of programmes: duration (short term (≤ 3 months) versus long term (over 3 months) [34], and formats such as mobile health (telephone, text message, mobile application, email and WeChat) versus face to face versus blended and providers (‘clinicians versus non-clinicians’ refers to individuals engaged in clinical work versus those who are not involved in direct patient care). Publication bias was performed when at least 10 studies were pooled for the same outcome through funnel plots and Egger’s regression test to assess asymmetry [35, 36].

### Sensitivity analysis

Leave-one-out sensitivity analysis was conducted by excluding one trial at a time to evaluate the stability of the pooled results [37].

## Results

### Search results

**Fig. 1:**
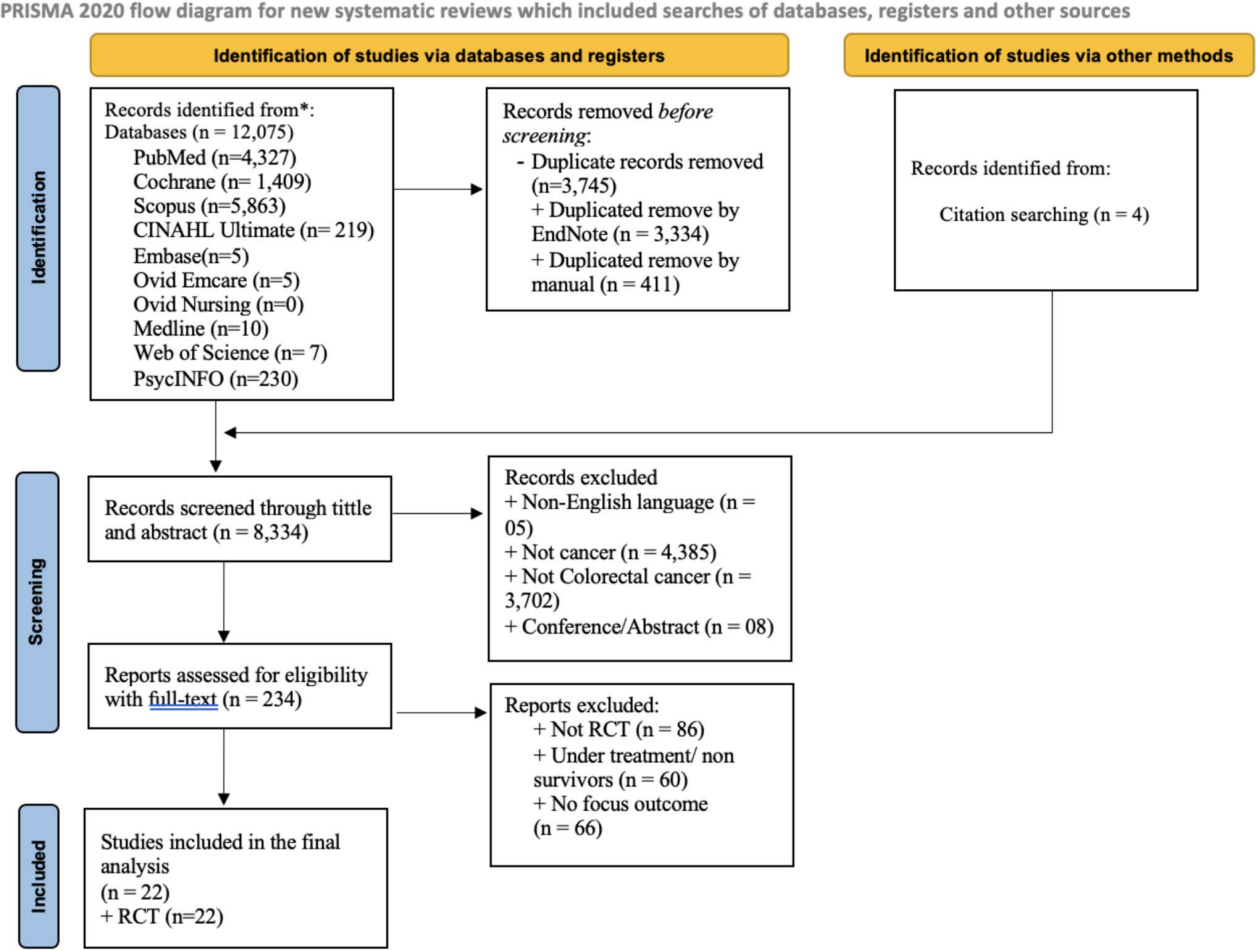
Study retrieval and selection flow chart (PRISMA). From: Page MJ, McKenzie JE, Bossuyt PM, Boutron I, Hoffmann TC, Mulrow CD, et al. The PRISMA 2020 statement: an updated guideline for reporting systematic reviews. BMJ 2021;372:n71. https://doi.org/10.1136/bmj.n71. For more information, visit: http://www.prisma-statement.org/

The database search yielded 12,075 articles and four articles for manual searching by citation-related search. After duplicates were removed, 8334 articles remained. The screening of titles and abstracts led to the exclusion of 8100 articles that did not meet the inclusion criteria. The full texts of the remaining 234 articles were then reviewed, resulting in the further exclusion of 212 articles. Ultimately, a total of 22 studies were included in the systematic review. Of these, 13 studies provided data for meta-analysis while the remaining 9 studies were not pooled quantitatively due to insufficient data and were synthesised narratively. The study retrieval and selection processes are presented in Fig. 1.

### Study characteristics

Table 1 shows the characteristics of 22 RCTs [38–59]. The studies were conducted in 12 developed countries/regions from 2003 to 2024: the USA (*N* = 5), Australia, Mainland China (*N* = 3 studies each), South Korea, Taiwan (*N* = 2 studies each), New Zealand, Denmark, Germany, the UK, Canada, Netherlands, Hong Kong SAR (*N* = 1 study each).

**Table 1:** The characteristic of the included studies

ID	Reference, country	Sample size	Age (mean)/cancer stage	Control group	Intervention				Outcome measures/scales
					Component	Mode/provider	Dose and duration	Follow-up	
1	Sun et al., 2024 USA	93	55.2 Ostomy, 6–24 months after treatment	Attention control	Promoting healthy behaviours	Individual, telephone Health coaches	27 min/session 10 sessions 17 weeks	9 weeks	Bowel function (MSK-BFI) Health-related quality of life (CHQoL-CRC)
2	Park and Lee, 2024 South Korea	58	56.9 N/A	Attention control	Management of long-term physical and psychological side effects	Individual, text messages Physician	20 min/session Twice weekly 12 weeks	3, 6, 12 months	Health-related quality of life (EORTC QLQ-C30)
3	Wang et al., 2023 Taiwan	156	62.3 Stage 1–2	Usual care	Promoting healthy behaviours	Individual, face to face Telephone Oncology nurses	Two 60-min session and 12 weekly follow-up call	6 months	Anxiety and depression symptom (HADS) Health-related quality of life (FACIT-F; FACT-C)
4	McCombie et at., 2023 New Zealand	68	67.3 Stage 1–4	Active control	Management of long-term physical and psychological side effects	Group, face to face Mindfulness teacher and psychologist	2 h/week 4 weeks	8 weeks, 6 months post-baseline	Anxiety and depression symptom (HADS) Health-related quality of life (SF-12)
5	Jefford et al., 2023 Australia	151	63.0 Stage 1–2	Usual care	Coordinating care	Individual, face to face Oncologist, physician	12 months	6 months, 12 months	Health-related quality of life (EORTC QLQ-C30)
6	Hovdenak et al., 2023 Denmark	336	65.2 Stage 0–3	Standard care	Surveillance and screening for the second cancer programme Management of long-term physical and psychological side effects	Face to face, telephone, email Nurses	Structured one-time patient-led programme	36 months	Health-related quality of life (FACT-C)
7	Wang et al., 2022 China	56	67.5 Stage 1–3	Routine care	Management of long-term physical and psychological side effects	WeChat/telephone Dietitians	6 months	6 months	Health-related quality of life (EORTC QLQ-C30)
8	Chan et al., 2022 USA	42	54.4 Stage 1–4	Active control	Promoting healthy behaviours	Fitbit wear, text message NA	2–3 times/week 150 min/session 12 weeks	12 weeks	Health-related quality of life (FACT-C) (SF-36)
9	Zhou and Sun, 2021 China	210	64.0 Stage 1–3	Attention care	Management of long-term physical and psychological side effects	Group, face to face Nurses	One session every two weeks 12 months	36 months	Anxiety and depression symptom (HADS) Health-related quality of life (EORTC QLQ-C30)
10	Zhang et al., 2020 China	159	60.8 Stage 1–4	Conventional care	Management of long-term physical and psychological side effects	Face to face Oncologist or Psychologist	On session every two week 3 months	3 months, 6 months	Health-related quality of life (EORTC QLQ-C30) Anxiety and depression symptom (HADS) Distress scale (CRD)
11	Yang et al., 2020 Taiwan	68	60.0 Ostomy, Stage 1–4	Attention care	Management of long-term physical and psychological side effects	Face to face Occupational therapist	15–30 min 1 month	1-month, 3 months	Health-related quality of life (WHOOQOL-BREF)
12	Ho et al., 2020 Hong Kong	223	65.2 Ostomy, Stage 1–4	Usual care	Promoting healthy behaviours	Face to face, telephone Dietitians and physicians	2 sessions, followed by phone call every 2 weeks 6 week to 6 months	12 months	Health-related quality of life (SF-12) Health-related quality of life (FACT-C) Anxiety and depression symptom (HADS)
13	Kim et al., 2019 Korea	71	56.2 Stage 2–3	Usual care	Promoting healthy behaviours	Face to face, telephone Exercise trainers	18 h/week during the first 6 weeks, increased to 27 h/week after 6 weeks 12 weeks	12 weeks	Health-related quality of life (FACT-C) Fatigue (FACIT-F)
14	Brown et al., 2018 USA	39	N/A Stage 1–3	Usual care	Promoting healthy behaviours	In‐person, telephone and email Physician	150–300 min per week 6 months	6 months	Health-related quality of life (SF‐36) Health-related quality of life (FACT-C) Fatigue (FSI39) Bowel dysfunction (NCCTG)
15	Jefford et al., 2016) Australia	221	62.1 Stage 1–3	Usual care	Coordinating care	Face to face, Telephone Nurses	60 min 8 weeks	2 months, 6 months	Distress scale (BSI-18) Health-related quality of life (EORTC QLQ-C30)
16	Cramer et al., 2016 Germany	54	68.3 Non-metastatic Stage 1–3	Usual care	Promoting healthy behaviours	Face to face Yoga instructor	90 min/week 10 weeks	10 weeks, 22 weeks	Health-related quality of life (FACT-C) Fatigue (FACIT-F) Anxiety and depression symptom (HADS)
17	Hawkes et al., 2014b , Australia	410	64.9 Non-metastatic stage 1–4	Usual care	Management of long-term physical and psychological side effects	Telephone Health coaches	11 sessions 6 months	6 months, 12 months	Health-related quality of life (FACT-C) Distress scale (BSI-18)
18	Pinto et al., 2013 USA	46	59.5 Stages 1–3	Usual care	Promoting healthy behaviours	Telephone Oncologist	5 days/week 30 min/session 12 weeks	3, 6 and 12 months	Fatigue (FACIT-F) Health-related quality of life (SF‐36) Health-related quality of life (FACT-C)
19	Bourke et al., 2011 UK	18	69 Stage 1–3	Standard care	Promoting healthy behaviours	Face to face Physiologist	2 sessions/week 12 weeks	12 weeks	Health-related quality of life (FACT-C) Fatigue (FACIT-F)
20	Courneya et al., 2003 Canada	102	60.0 N/A	Usual care	Promoting healthy behaviours	Telephone Fitness consultant	3–5 times/weeks 20–30 min/session 16 weeks	16 weeks	Health-related quality of life (FACT-C) Anxiety and depression (CESD) Fatigue (FACIT-F)
21	Mayer et al., 2018 USA	284	57.8 Stage 1–3	Attention care	Management of long-term physical and psychological side effects Promoting healthy behaviours	Apps Coach	150 min/week 3 months	3,6, 9 months	Health-related quality of life (FACT-C) Distress (NCCN)
22	Custers et al., 2024 Netherlands	84	63.7 Stage 1–3	Usual care	Management of long-term physical and psychological side effects	Website, telephone Psychologists	8 sessions 15–60 min/session 14 weeks	7 months	Health-related quality of life (EORTC QLQ-C30) Anxiety and depression symptom (HADS) Fatigue (CIS)

*BSI-18* The Brief Symptom Inventory 18, *CESD* The Centre for Epidemiological Studies Depression scale, *CHQoL-CRC* The City of Hope Quality of Life-Colorectal Cancer questionnaire, *CIS* The 20-item Checklist Individual Strength, *CRD* Cancer-related distress, *EORTC QLQ-C30* The European Organization for Research and Treatment of Cancer Quality of Life Scale, *FACIT-F* Functional Assessment of Chronic Illness Therapy—Fatigue, *FSI39* The Fatigue Symptom Inventory, *HADS* The Hospital Anxiety and Depression Scale, *MSK-BFI* The bowel function scale score, *NCCN* The National Comprehensive Cancer Network distress tool, *NCCTG* The North Central Cancer Treatment Group questionnaire, *SF-12* The Short Form-12, *SF-36* The Medical Outcomes Survey Short Form, *WHOOQOL-BREF* The World Health Organization Quality of Life

All studies included a total of 2949 CRC survivors with sample sizes ranging from 18 to 410 participants. The average age of the survivors varied from 54.4 to 68.26 years. Three studies reported that the survivors had an ostomy following treatment. Two studies reported that participants were in the early stages 1–2 of the disease, eleven in stage 3 and five in stage 4 (late-stage with metastasis).

### Interventions

Based on the guidelines suggested by the ACS, four main components of the survivorship programmes were identified (Table S1). Most of the survivorship programmes (*N* = 11) in the included studies focus on promoting health behaviours, such as exercises and a healthy diet. Eight studies explored various forms of aerobic exercise, resistance training, walking, cycling, yoga and core stabilisation exercises, which were conducted under supervision or in a home-based setting [46, 51–53, 55, 57–59]. Additionally, two studies provided dietary interventions that included structured diet regimens, such as reductions in red and processed meat intake and low-fat diets, along with dietary counselling [38, 45]. One study combined exercise and nutritional interventions [50].

Furthermore, many programmes (*N* = 9) emphasised managing long-term physical and psychological side effects by educational and psychological interventions. For education, two studies offered colostomy education with self-management techniques, potential late effects and alarm symptoms [41, 44] while three studies provided healthy lifestyle education related to work and wellness [39, 49, 52]. For psychological intervention, one study utilised acceptance and commitment techniques for psychoeducation [56], and three studies implemented psychological interventions that incorporated music, guided imagery and cognitive restructuring to reduce stress and control emotion [48]; mindfulness [42]; and psychoeducation, cognitive restructuring, behaviour modification and relaxation [40]. Additionally, one study used reminiscence therapy to help individuals express their thoughts and feelings about cancer [47].

Two of the programmes promoted care coordination. One study involved follow-up care shared between hospital-based oncologists and survivors’ general physicians to monitor CRC recurrence after discharge, instead of traditional hospital-based follow-up care [43]. Another study provided a survivorship care plan that included tailored information on treatment and follow-up recommendations from health providers [54]. However, only one study focused on CRC surveillance and screening for second cancer, such as educating survivors about the signs of new cancers [44].

#### Delivery mode

Seven programmes were conducted face to face. Six studies utilised social media platforms for support, including WeChat, application, email and text messages. Six studies employed a combination of face to face and telephone, and three studies were delivered solely through telephone.

#### Duration

In terms of programme duration, six studies lasted from 6 months to 3 years, three studies lasted for 16 weeks, seven studies were conducted for 12 weeks, five were conducted from 4 to 10 weeks and 15 studies conducted the follow-up after post-treatment. Each programme ranged from 4 to 24 sessions, and the frequency between sessions averaged weekly to monthly.

#### Deliverer

Four studies were delivered by nurses; five by oncologists/physicians; eleven by health-related professionals, such as occupational therapists, psychologists, dietitians, fitness trainers and yoga instructors; and one study combined physicians and dietitians.

#### Measurements

This review included six different scales to assess various types of HRQoL: the City of Hope Quality of Life-Colorectal Cancer questionnaire (*N* = 1), the Functional Assessment of Cancer Therapy-Colorectal (FACT-C, *N* = 12), the European Organization for Research and Treatment of Cancer Quality of Life Scale (EORTC QLQ-C30, *N* = 7), the Short Form-12 (SF-12, *N* = 2), the World Health Organization Quality of Life (WHOOQOL-BREF, *N* = 1) and the Medical Outcomes Survey Short Form (SF‐36, *N* = 3). It also included other scales, including distress (*N* = 3), depression (*N* = 2), anxiety (*N* = 1), bowel dysfunction (*N* = 2) and fatigue (*N* = 2).

### Methodological quality

**Fig. 2:**
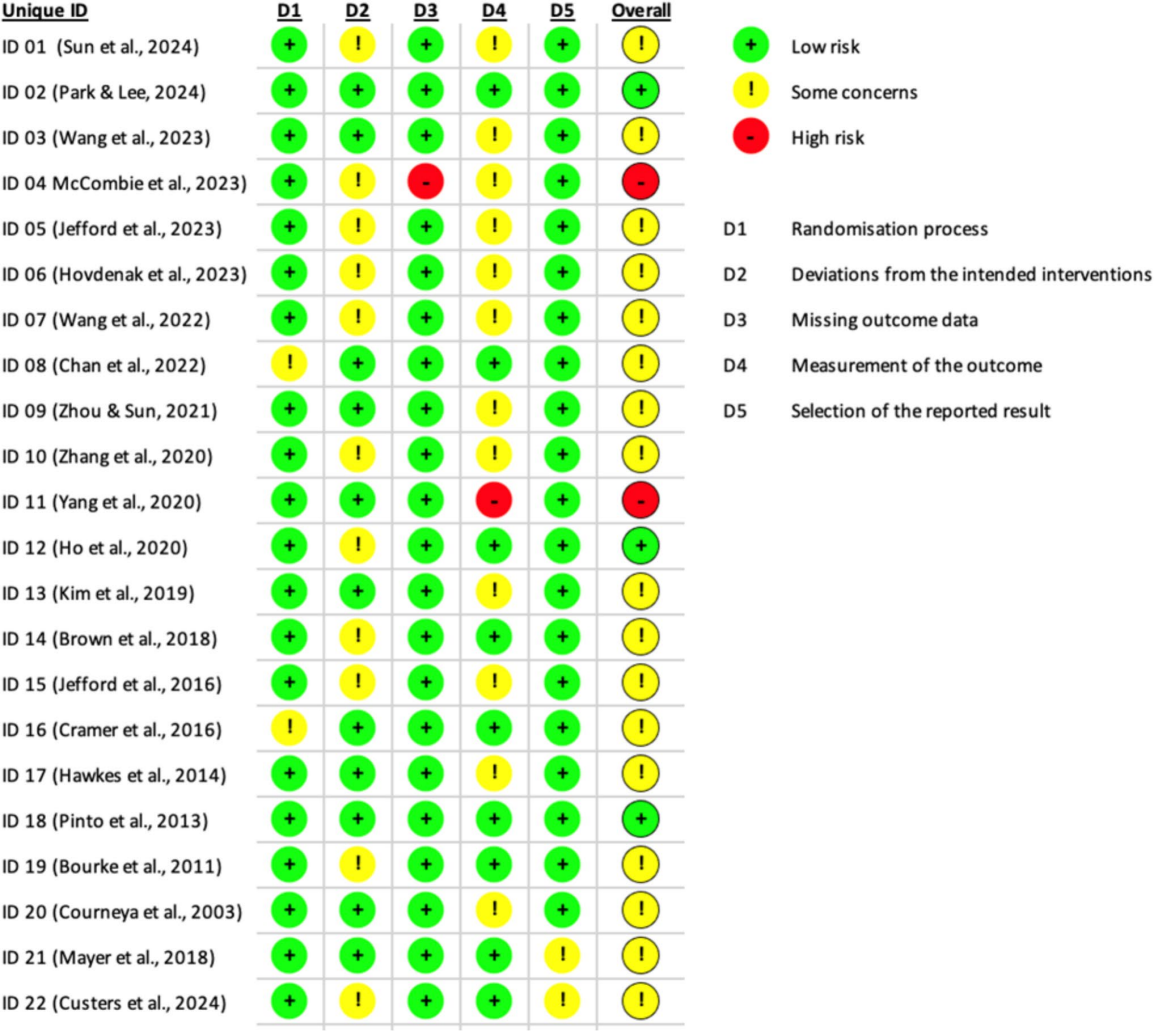
Risk-of-Bias 2.0 (RoBs)

All studies consistently measured outcomes for the experimental groups and adhered to the correct statistical analyses and trial designs. Two studies were assessed as ‘high risk’ due to deviations from missing outcome data [42] and measurement of the outcome [49]. Other studies raised ‘some concerns’ regarding insufficient information on allocation concealment, non-blindness of intervention providers and outcome assessors. All studies reported sufficient information on predetermined data analysis plans or protocols. Overall, the studies ranged from having some concerns to a high risk of bias in methodological quality (Fig. 2).

The quality of the studies included in this review was evaluated using the GRADE system. The quality of evidence for the HRQoL outcome was downgraded to the range of ‘very low’ and ‘low’ because of the risk of bias, high heterogeneity and imprecision, whereas that of other outcomes was from ‘low’ to ‘moderate’ (Supplementary File 3).

### Publication bias

**Fig. 3:**
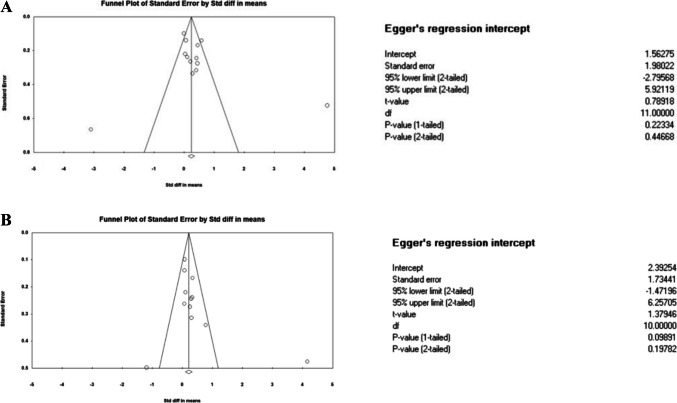
Publication bias; **A** HRQoL_(Physical). **B** HRQoL_(Mental)

No publication bias was found for the included studies with physical and mental HRQoL outcomes, with Egger’s test results of *t* = 0.789, df = 11 and *P* = 0.446 and *t* = 1.379, df = 10 and *P* = 0.197, respectively (Fig. 3).

### Effects of survivorship programmes on health-related QoL

#### HRQoL

**Fig. 4:**
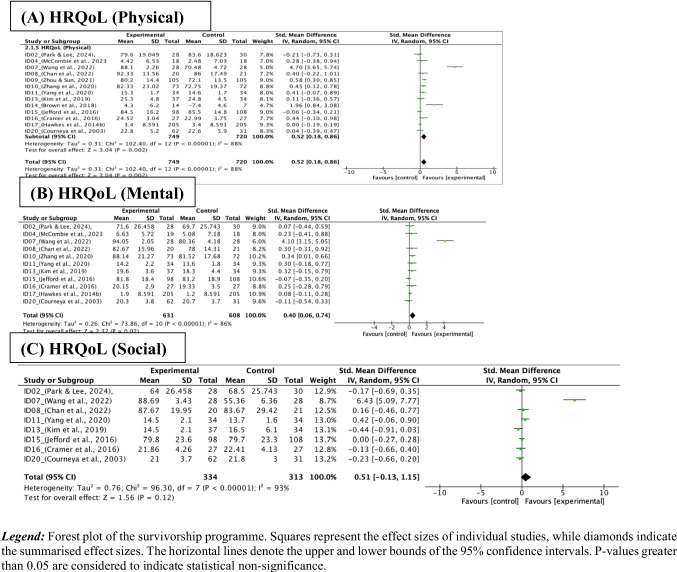
Forest plot of the survivorship programme comparing the survivorship programme (experimental) with the usual care (control) in health-related quality of life outcome. Legend: Forest plot of the survivorship programme. Squares represent the effect sizes of individual studies, while diamonds indicate the summarised effect sizes. The horizontal lines denote the upper and lower bounds of the 95% confidence intervals. *P*-values greater than 0.05 are considered to indicate statistical non-significance

The meta-analysis results indicated a significant improvement in HRQoL related to physical (13 studies with 1469 CRC survivors) [39, 42, 45–49, 51, 53–56, 59] and mental (11 studies with 1239 CRC survivors) [39, 42, 45, 46, 48, 49, 51, 54–56, 59] domains following the survivorship programmes: (SMD = 0.52, 95% CI [0.18, 0.86], *P* = 0.002, *I*^2^ = 88%) and (SMD = 0.4, 95% CI [0.06, 0.74], *P* = 0.02, *I*^2^ = 86%), respectively (Fig. 4A and B). However, a non-significant improvement was observed in HRQoL related to social domain (08 studies with 647 CRC survivors) [39, 45, 46, 49, 51, 54, 55, 59] (SMD = 0.51, 95% CI [−0.13, 1.15], *P* = 0.12, *I*^2^ = 93%), as shown in Fig. 4C. Amongst the 22 studies, nine reported insufficient data for meta-analysis due to omitting the standard deviation or reporting selective statistics only, such as adjusted mean differences, slope coefficients and *P*-values. Narrative synthesis was conducted to assess the effectiveness of this outcome. Seven studies showed no significant differences in HRQoL indicators, suggesting that the control group had similar features to the intervention to promote exercise, diet and psychological well-being, leading to no significant difference [38, 43, 44, 50, 52, 57, 58]. Meanwhile, one study demonstrated that the intervention group achieved significantly higher HRQoL scores compared to the control group immediately post-intervention (*P* = 0.012) [41], while another noted improvement in the intervention group after 7 months [40].

Sensitivity analysis, which involved the removal of the heterogeneous study [45], had distinct effects. For the HRQoL in mental domain, heterogeneity was eliminated (*P* = 0.04, *I*^2^ = 0%). For the HRQoL in physical domain, its removal reduced the heterogeneity from high to moderate (*P* = 0.01, *I*^2^ = 65%), as shown in Supplementary File 4. Sensitivity analysis was also performed by removing two studies with a high risk of bias, and this made no significant difference to the overall results.

Subgroup analyses were conducted to examine the duration, formats and providers of the programme with HRQoL in physical and mental domains. Notably, the subgroup identified significant difference for duration, with long-term programmes (over 3 months) showing a greater effect on physical HRQoL than short-term programmes (≤ 3 months, *P* = 0.02). For providers, clinician-led programmes demonstrated a significantly greater effect on physical HRQoL than non-clinician-led programmes (*P* = 0.01). By contrast, whilst the subgroup differences were non-significant across the formats (*P* = 0.66 for physical and *P* = 0.29 for mental), the mHealth format was associated with the largest effect size compared with the face to face and blended formats.

#### Distress, anxiety and depression

Three studies tested distress in 287 CRC survivors [40, 42, 48], eight studies measured anxiety in 880 CRC survivors [40–42, 47, 48, 50, 55, 59] and nine studies measured depression in 960 CRC survivors [40–42, 47, 48, 50, 51, 55, 59]. These studies were pooled and included in the meta-analysis. Significant effects were found for symptoms of distress (SMD = −0.55, 95% CI [−0.78, −0.31], *P* < 0.0001, *I*^2^ = 0%), anxiety (SMD = −0.35, 95% CI [−0.53, −0.17], *P* = 0.0001, *I*^2^ = 41%) and depression (SMD = −0.23, 95% CI [−0.36, −0.1], *P* = 0.0004, *I*^2^ = 0%), as shown in Supplementary Files 5A–C).

#### Fatigue

Eight studies that measured fatigue in 755 CRC survivors were pooled and included in the meta-analysis [40, 41, 44, 51, 55, 57–59]. No significant effects were found for fatigue (SMD = 0.007, 95% CI [−0.07, 0.22], *P* = 0.31, *I*^2^ = 0%; Supplementary File 5D).

#### Bowel function

Two studies that measured bowel function in 338 CRC survivors were pooled and included in the meta-analysis [38, 44]. No significant effects were found for bowel function (SMD = −0.01, 95% CI [−0.35, 0.32], *P* = 0.93, *I*^2^ = 51%; Supplementary File 5E).

## Discussion

This systematic review and meta-analysis was conducted to assess the effectiveness of survivorship programmes for improving post-treatment HRQoL in CRC survivors. A total of 22 RCTs involving 2949 CRC survivors were included. The meta-analysis results (13 studies) indicated that the programmes had a significant positive effect on the physical and mental domains of HRQoL and on anxiety, depression and distress. However, no significant effects were found for the social domain of HRQoL, fatigue and bowel function.

This meta-analysis found that these programmes did not have significant effects on the social domain of HRQoL, which may be associated with finances, relationships, social activities or work performance post-treatment, because such factors are often perceived differently by each individual [60, 61]. The mean age of CRC survivors in the present study ranged from 54.4 to 68.26 years, highlighting a distinct difference between the two groups: the working group (60 years or younger) and the retired group (older 60 years). This division strengthened the varying social needs of each group. The former group often faced pressures related to work or finances, whereas the latter concentrated on family relationships and personal interests. Besides, the characteristic heterogeneity amongst CRC survivors in the present study, including ostomy status, stage and metastasis, is likely a confounding factor that influenced the reported HRQoL scores. In addition, the timing of HRQoL score assessment in the programmes varies, as well as the use of different scales. This difference contributes to the high heterogeneity observed in this review, underscoring the need for rigorous methodological research to evaluate the effectiveness of such programmes moving forward.

This review identified key programme components for improving HRQoL in CRC survivors. This finding indicated a heterogeneous service delivery model for CRC survivors. The meta-analysis results for subgroups revealed that the survivorship programmes provided by clinicians had a greater effect on HRQoL than those led by non-clinicians. Besides, amongst clinician-led providers, studies have emphasised the vital role of nurses in supporting CRC survivors. One key finding is that patients expect nurses to provide information about the disease and treatment-related side effects, offer hope and advocate on their behalf [62]. Because nurses also serve as educators, coordinators, monitoring providers and palliative care providers [63]. Many reported enhanced satisfaction with their care and a sense of safety post-discharge due to continued contact with nurses who facilitated their transition [64]. Given these insights, future studies on survivorship programmes led by nurses are warranted to further explore and enhance the support provided to this vulnerable group.

The subgroup meta-analysis indicated that the improvements in physical HRQoL are more pronounced in the long term (> 3 months) rather than the short term as prior reported [28]. Previous research demonstrated that HRQoL scores tend to improve after 4 months and continue thereafter [65, 66]. However, if an approach that includes monitoring survivors’ adherence is adopted for interventions, the scores may improve within the initial 3 months [67]. Furthermore, our analysis revealed that mHealth-based programmes yielded a more substantial effect on HRQoL than face to face or blended formats. This finding underscores the pivotal role of technology in enhancing the patient-provider relationship and aligns with previous meta-analyses reporting significant HRQoL improvements among breast cancer survivors utilising mobile health platforms [68]. The interventions in this review leveraged diverse platforms, including mobile applications, SMS, websites, WeChat and wearable devices like Fitbit, to facilitate education and treatment adherence. Such mHealth formats appear uniquely suited for cancer survivors post-discharge, providing flexible and accessible support during the transition to recovery [69]. However, despite these benefits, rapid disengagement and declining adherence remain critical barriers [70]. Future research should prioritise a co-design approach to develop mHealth solutions tailored to survivors’ specific preferences and real-world needs, thereby ensuring sustained engagement and long-term effectiveness. Notably, the demographic data in the present study were exclusively from CRC survivors in developed countries, highlighting a significant lack of geographic diversity, especially given the increasing prevalence of CRC in developing nations [71]. The implementation of mHealth in these regions should be approached with caution, as its effectiveness is closely tied to cultural factors. For example, many survivors may be more familiar with traditional healthcare services with face-to-face consultations than mHealth solutions. Hence, more research in developing countries is urgently needed to explore mHealth-based survivorship programmes to optimise individual survivorship and enhance HRQoL.

## Study strengths and limitations

This systematic review and meta-analysis of RCTs contributes to the evidence on the details of components and the effectiveness of survivorship programmes in enhancing HRQoL in CRC survivors followed the ACS guidelines. However, this review has several limitations. Firstly, the inclusion of English-language publications only may have introduced a geographic bias, thus limiting the generalisability of the findings beyond predominantly English-speaking regions. Secondly, the exclusion of conference abstracts, dissertations and other grey literature may have resulted in selection bias. Thirdly, the overall methodological quality of the included studies was rated as having ‘some concerns’, which should be considered when interpreting the results. However, both the primary meta-analysis and sensitivity analysis, performed after excluding two studies at high risk of bias, indicate that the survivorship programmes were more effective than usual care. Therefore, the inclusion of studies at high risk of bias is unlikely to have influenced the overall findings. Finally, all included studies measured HRQoL, and this focus may have led to the underreporting of other relevant outcomes. However, this meta-analysis not only reported HRQoL outcomes but also substantially included psychological and physical symptoms that are known to influence HRQoL. Therefore, healthcare providers could consider this study as a viable option to improve comprehensive health outcomes for CRC survivors.

## Implications for practice

This systematic review and meta-analysis provides important evidence for the design of survivorship programmes for CRC survivors. Future studies should control for potential confounders, including differences in demographics and culture, which may influence intervention effectiveness in diverse populations, especially in developing countries. Furthermore, the frequency and format of intervention (e.g. mHealth) should be carefully considered given their association with health behaviours and barriers to mHealth use. The optimal approach is through a co-design, where healthcare providers, developers and CRC survivors (end-users) may collaborate closely to develop mHealth-based programmes. This approach ensures that programmes are personalised to specific needs, considers sociodemographic factors and can be effectively integrated into local health systems.

## Conclusion

This systematic review evaluated the survivorship programmes effectively improved HRQoL and reduced psychological. Components of such programmes include education, psychological intervention, exercise, diet programmes and survivorship care plan. Moreover, a nurse-led approach combined with the mHealth format post-treatment is expected to incorporate the four elements of ACS. Future studies that integrate these elements into mHealth initiatives to help CRC survivors remotely access timely medical care are warranted.

## Supplementary information

Below is the link to the electronic supplementary material.

ESM 1 (DOCX 979 KB)

## Data Availability

The datasets used for this study are available from the corresponding authors on reasonable request.
