# Supplementary material for: Effectiveness of survivorship programmes to enhance health-related quality of life of colorectal cancer survivors: a systematic review and meta-analysis of randomised controlled trials
Source: Support Care Cancer. 2026 May 18;34(6):551. doi: 10.1007/s00520-026-10779-8 (PMC13183737; doi:10.1007/s00520-026-10779-8)
Supplement: Supplementary file 1 — (DOCX 979 KB) [file 520_2026_10779_MOESM1_ESM.docx]

**Supplementary Material**

**Effectiveness of survivorship programme to enhance health-related quality of life of colorectal cancer survivors: A systematic review and meta-analysis of randomised controlled trials**

**Summary**

[Supplementary File 1. Details of Inclusion and exclusion criteria 2](#_Toc215215461)

[Supplementary File 2. Full search strategy for each database 3](#_Toc215215462)

[Supplementary File 3. The Grading of Recommendation, Assessment, Development, and Evaluation (GRADE) system 6](#_Toc215215463)

[Supplementary File 4. Sensitivity analysis 8](#_Toc215215464)

[Supplementary File 5. Forest plot of the survivorship programme comparing the survivorship programme (experimental) with the usual care (control) in secondary outcome. 9](#_Toc215215465)

# **Supplementary File 1.** Details of Inclusion and exclusion criteria

| ***Population (P):*** | CRC survivors (18 years of age and older) who were clinically diagnosed with CRC and completed primary treatment (surgery and/or chemotherapy and/or radiation) were included. Studies that included those who were undergoing treatment/adjunct treatment were excluded. |
| --- | --- |
| ***Intervention (I):*** | A survivorship programme is defined as any intervention carried out for CRC survivors in the survivorship phase, provided that it aligns with at least one of the four dimensions outlined in the ACS recommendations. This means that included studies need to satisfy one of the following four criteria: 1) surveillance of CRC recurrence and screening for second primary cancers, 2) assessment and management of the long-term physical and psychological effects of CRC and its treatment, 3) promotion of healthy behaviours or 4) coordination of care between specialists and primary care physicians. |
| ***Comparison (C ):*** | Participants in the comparison group should have received standard or usual care; participants receiving different kinds of interventions should have been compared with those receiving survivorship programmes in the intervention group. |
| ***Outcomes of interest (O):*** | All studies that addressed the HRQoL of CRC survivors were included in this review. The secondary outcomes of interest were distress, depression, anxiety, fatigue and bowel dysfunction because these are symptoms commonly reported by CRC survivors and influencers of their HRQoL. |
| ***Study design (S):*** | Only RCTs, including pilot RCTs, were included. |

# **Supplementary File 2.** Full search strategy for each database

| **No.** | **Databases** | **Keywords** | | **Results** | |
| --- | --- | --- | --- | --- | --- |
| 1 | PubMed | (((colon[Title/Abstract] OR rectal[Title/Abstract] OR colorectal[Title/Abstract] OR bowel[Title/Abstract] OR cancer[Title/Abstract] OR neoplasm[Title/Abstract] OR oncology[Title/Abstract] OR malign*[Title/Abstract] OR tumour[Title/Abstract] OR tumor[Title/Abstract] OR survivors[Mesh] OR colorectal cancer survivors[Title/Abstract]) AND (survivorship[Mesh] OR post- treatment[Title/Abstract] OR follow- up[Title/Abstract] OR after treatment[Title/Abstract] OR intervention[Title/Abstract] OR programme[Title/Abstract] OR programmeme[Title/Abstract])) AND (Randomized Controlled Trial[Title/Abstract] OR random[Title/Abstract] OR RCT[Title/Abstract])) AND (Quality of life[Title/Abstract] OR Health-related quality of life[Title/Abstract] OR value of life[Title/Abstract] OR QoL[Title/Abstract] OR depression[Title/Abstract] OR anxiety[Title/Abstract] OR distress[Title/Abstract] OR psychology[Title/Abstract] OR bowel dysfunction[Title/Abstract] OR bowel function[Title/Abstract]),,,"(""colon""[Title/Abstract] OR ""rectal""[Title/Abstract] OR ""colorectal""[Title/Abstract] OR ""bowel""[Title/Abstract] OR ""cancer""[Title/Abstract] OR ""neoplasm""[Title/Abstract] OR ""oncology""[Title/Abstract] OR ""malign*""[Title/Abstract] OR ""tumour""[Title/Abstract] OR ""tumor""[Title/Abstract] OR ""survivors""[Title/Abstract] OR ""colorectal cancer survivors""[Title/Abstract]) AND (""survivorship""[Title/Abstract] OR ""post treatment""[Title/Abstract] OR ""follow up""[Title/Abstract] OR ""after treatment""[Title/Abstract] OR ""intervention""[Title/Abstract] OR ""programme""[Title/Abstract] OR ""programmeme""[Title/Abstract]) AND (""randomized controlled trial""[Title/Abstract] OR ""random""[Title/Abstract] OR ""RCT""[Title/Abstract]) AND (""quality of life""[Title/Abstract] OR ""health related quality of life""[Title/Abstract] OR ""value of life""[Title/Abstract] OR ""QoL""[Title/Abstract] " | | 4,327 | |
| 2 | Cochrane | 1 | (colon OR rectal OR colorectal OR bowel OR cancer OR neoplasm OR oncology OR malign* OR tumour OR tumor OR survivors OR colorectal cancer survivors):ti (Word variations have been searched) | 170683 | 1409 |
|  |  | 2 | ((survivorship OR post-treatment OR follow-up OR after treatment) AND (intervention OR programme OR programmeme)):ab (Word variations have been searched) | 221951 |  |
|  |  | 3 | (Randomized Controlled Trial OR random OR RCT):ab (Word variations have been searched) | 1074506 |  |
|  |  | 4 | (Quality of life OR Health-related quality of life OR value of life OR QoL):ti (Word variations have been searched) | 91098 |  |
|  |  | 5 | #1 AND #2 AND #3 AND #4 | 1409 |  |
| 3 | Scopus | 1 | TITLE-ABS-KEY ( colon OR rectal OR colorectal OR bowel OR cancer OR neoplasm OR oncology OR malign* OR tumour OR tumor OR survivors OR "colorectal cancer survivors | [6,881,252](https://www.scopus.com/search/history/results.uri?origin=searchhistory&shid=1) | 5,863 |
|  |  | 2 | TITLE-ABS-KEY ( ( survivorship OR "post-treatment" OR "follow-up" OR "after treatment" ) ) | 2,635,387 |  |
|  |  | 3 | TITLE-ABS-KEY ( intervention OR programme OR programmeme ) | 4,998,074 |  |
|  |  | 4 | ( TITLE-ABS-KEY ( ( survivorship OR "post-treatment" OR "follow-up" OR "after treatment" ) ) ) AND ( TITLE-ABS-KEY ( intervention OR programme OR programmeme ) ) | 371,926 |  |
|  |  | 5 | TITLE-ABS-KEY ( "randomized controlled trial" OR random OR rct ) | 2,479,418 |  |
|  |  | 6 | TITLE-ABS-KEY ( "quality of life" OR "health-related quality of life" OR "value of life" OR qol) | 2,944,482 |  |
|  |  | 7 | #1 AND #4 AND #5 AND #6 | 5,863 |  |
| 4 | CINAHL Ultimate | 1 | (colon OR rectal OR colorectal OR bowel OR cancer OR neoplasm OR oncology OR malign* OR tumour OR tumor OR survivors OR colorectal cancer survivors):ti (Word variations have been searched) | 529.015 | 219 |
|  |  | 2 | ((survivorship OR post-treatment OR follow-up OR after treatment) AND (intervention OR programme OR programmeme)):ab (Word variations have been searched) | 84.890 |  |
|  |  | 3 | (Randomized Controlled Trial OR random OR RCT):ab (Word variations have been searched) | 168.653 |  |
|  |  | 4 | (Quality of life OR Health-related quality of life OR value of life OR QoL):ti (Word variations have been searched) | 165.456 |  |
|  |  | 5 | #1 AND #2 AND #3 AND #4 | 219 |  |
| 5 | Embase | 1 | (colon OR rectal OR colorectal OR bowel OR cancer OR neoplasm OR oncology OR malign* OR tumour OR tumor OR survivors OR colorectal cancer survivors):ti | 2.683.124 | 5 |
|  |  | 2 | ((survivorship OR post-treatment OR follow-up OR after treatment) AND (intervention OR programme OR programmeme)):ti | 4.826 |  |
|  |  | 3 | (Randomized Controlled Trial OR random OR RCT):ti | 108.318 |  |
|  |  | 4 | (Quality of life OR Health-related quality of life OR value of life OR QoL):ti | 431.312 |  |
|  |  | 5 | #1 AND #2 AND #3 AND #4 | 5 |  |
| 6 | Ovid Emcare | 1 | (colon OR rectal OR colorectal OR bowel OR cancer OR neoplasm OR oncology OR malign* OR tumour OR tumor OR survivors OR colorectal cancer survivors):ti | 492899 | 5 |
|  |  | 2 | ((survivorship OR post-treatment OR follow-up OR after treatment) AND (intervention OR programme OR programmeme)):ti | 1724 |  |
|  |  | 3 | (Randomized Controlled Trial OR random OR RCT):ti | 42970 |  |
|  |  | 4 | (Quality of life OR Health-related quality of life OR value of life OR QoL):ti | 165288 |  |
|  |  | 5 | #1 AND #2 AND #3 AND #4 | 5 |  |
| 7 | Ovid Nursing | 1 | (colon OR rectal OR colorectal OR bowel OR cancer OR neoplasm OR oncology OR malign* OR tumour OR tumor OR survivors OR colorectal cancer survivors):ti | 14086 | 0 |
|  |  | 2 | ((survivorship OR post-treatment OR follow-up OR after treatment) AND (intervention OR programme OR programmeme)):ti | 111 |  |
|  |  | 3 | (Randomized Controlled Trial OR random OR RCT):ti | 2888 |  |
|  |  | 4 | (Quality of life OR Health-related quality of life OR value of life OR QoL):ti | 7325 |  |
|  |  | 5 | #1 AND #2 AND #3 AND #4 | 0 |  |
| 8 | Medline | 1 | (colon OR rectal OR colorectal OR bowel OR cancer OR neoplasm OR oncology OR malign* OR tumour OR tumor OR survivors OR colorectal cancer survivors):ti | 2,238,212 | 10 |
|  |  | 2 | ((survivorship OR post-treatment OR follow-up OR after treatment) AND (intervention OR programme OR programmeme)):ti | 4,300 |  |
|  |  | 3 | (Randomized Controlled Trial OR random OR RCT):ti | 140,647 |  |
|  |  | 4 | (Quality of life OR Health-related quality of life OR value of life OR QoL):ti | 336,893 |  |
|  |  | 5 | #1 AND #2 AND #3 AND #4 | 10 |  |
| 9 | Web of Science | 1 | (colon OR rectal OR colorectal OR bowel OR cancer OR neoplasm OR oncology OR malign* OR tumour OR tumor OR survivors OR colorectal cancer survivors):ti | 2,836,448 | 7 |
|  |  | 2 | ((survivorship OR post-treatment OR follow-up OR after treatment) AND (intervention OR programme OR programmeme)):ti | 6,74 |  |
|  |  | 3 | (Randomized Controlled Trial OR random OR RCT):ti | 342,324 |  |
|  |  | 4 | (Quality of life OR Health-related quality of life OR value of life OR QoL):ti | 564,109 |  |
|  |  | 5 | #1 AND #2 AND #3 AND #4 | 7 |  |
| 10 | PsycINFO | 1 | (colon or rectal or colorectal or bowel or cancer or neoplasm or oncology or malign* or tumour or tumor or survivors or colorectal cancer survivors).mp. [mp=title, abstract, heading word, table of contents, key concepts, original title, tests & measures, mesh word] | 144855 | 230 |
|  |  | 2 | (survivorship or post-treatment or follow-up or after treatment).mp. [mp=title, abstract, heading word, table of contents, key concepts, original title, tests & measures, mesh word] | 200985 |  |
|  |  | 3 | (intervention or programme or program).mp. [mp=title, abstract, heading word, table of contents, key concepts, original title, tests & measures, mesh word] | 628492 |  |
|  |  | 4 | #2 AND #3 | 57833 |  |
|  |  | 5 | (Randomized Controlled Trial or random or RCT).mp. [mp=title, abstract, heading word, table of contents, key concepts, original title, tests & measures, mesh word] | 117848 |  |
|  |  | 6 | (Quality of life or Health-related quality of life or value of life or QoL).mp. [mp=title, abstract, heading word, table of contents, key concepts, original title, tests & measures, mesh word] | 119722 |  |
|  |  | 7 | #1 AND #4 AND #5 AND #6 | 230 |  |
| 11 | TOTAL | | | | 12075 |

**Supplementary File 3.** The Grading of Recommendation, Assessment, Development, and Evaluation (GRADE) system

| **Certainty assessment** | | | | | | | | **№ of patients** | | **Effect** | | **Certainty** | **Importance** |
| --- | --- | --- | --- | --- | --- | --- | --- | --- | --- | --- | --- | --- | --- |
| **№ of studies** | **Study design** | **Risk of bias** | **Inconsistency** | **Indirectness** | **Imprecision** | **Other considerations** | **[intervention] Survivorship Programme** | | **[comparison] Usual care** | **Relative (95% CI)** | **Absolute (95% CI)** |  |  |
| **Health-reated QoL (Physical domain)** | | | | | | | | | | | | | |
| 13 | randomised trials | serious^a^ | serious^b^ | not serious | not serious | none | 749 | | 720 | - | SMD **0.52 SD higher** (0.18 higher to 0.86 higher) | ⨁⨁◯◯ Low^a,b^ |  |
| **Health-reated QoL (Mental domain)** | | | | | | | | | | | | | |
| 11 | randomised trials | serious^a^ | serious^b^ | not serious^b^ | not serious | none | 631 | | 608 | - | SMD **0.4 SD higher** (0.06 higher to 0.74 higher) | ⨁⨁◯◯ Low^a,b^ |  |
| **Health-reated QoL (Social domain)** | | | | | | | | | | | | | |
| 8 | randomised trials | serious^a^ | serious^a^ | not serious^b^ | serious^c^ | none | 334 | | 313 | - | SMD **0.51 SD higher** (0.13 lower to 1.15 higher) | ⨁◯◯◯ Very low^a,b,c^ |  |
| **Distress** | | | | | | | | | | | | | |
| 3 | randomised trials | not serious | not serious | not serious | serious^c^ | none | 144 | | 143 | - | SMD **0.55 SD fewer** (0.78 fewer to 0.31 fewer) | ⨁⨁⨁◯ Moderate^c^ |  |
| **Depression** | | | | | | | | | | | | | |
| 9 | randomised trials | serious^a^ | not serious | not serious | not serious | none | 500 | | 460 | - | SMD **0.23 SD fewer** (0.36 fewer to 0.1 fewer) | ⨁⨁⨁◯ Moderate^a^ |  |
| **Anxiety** | | | | | | | | | | | | | |
| 8 | randomised trials | serious^a^ | not serious | not serious | not serious | none | 456 | | 424 | - | SMD **0.35 SD fewer** (0.53 fewer to 0.17 fewer) | ⨁⨁⨁◯ Moderate^a^ |  |
| **Fatigue** | | | | | | | | | | | | | |
| 8 | randomised trials | not serious | not serious | not serious | serious^c^ | none | 390 | | 365 | - | SMD **0.07 SD higher** (0.07 lower to 0.22 higher) | ⨁⨁⨁◯ Moderate^c^ |  |
| **Bowel dysfunction** | | | | | | | | | | | | | |
| 2 | randomised trials | not serious | serious^b^ | not serious | serious^c^ | none | 172 | | 166 | - | SMD **0.01 SD lower** (0.35 lower to 0.32 higher) | ⨁⨁◯◯ Low^b,c^ |  |

**CI:** confidence interval; **SMD:** standardised mean difference

#### Explanations

a. Downgrade one level for risk of bias because including studies with high-risk

b. Downgrade one level for inconsistency because of high heterogeneity

c. Downgrade one level for imprecision because the sample size sufficient for one adequately powered study (min 400 people (continuous))

# **Supplementary File 4.** Sensitivity analysis

**(A) HRQoL (Physical)**

| **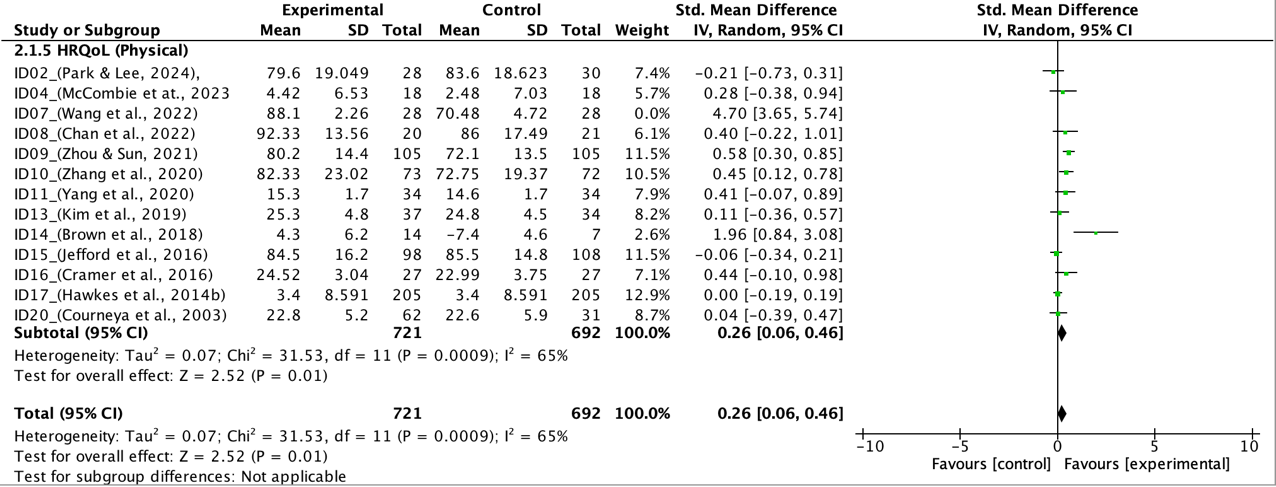**  **(B) HRQoL (Mental)** |
| --- |
| **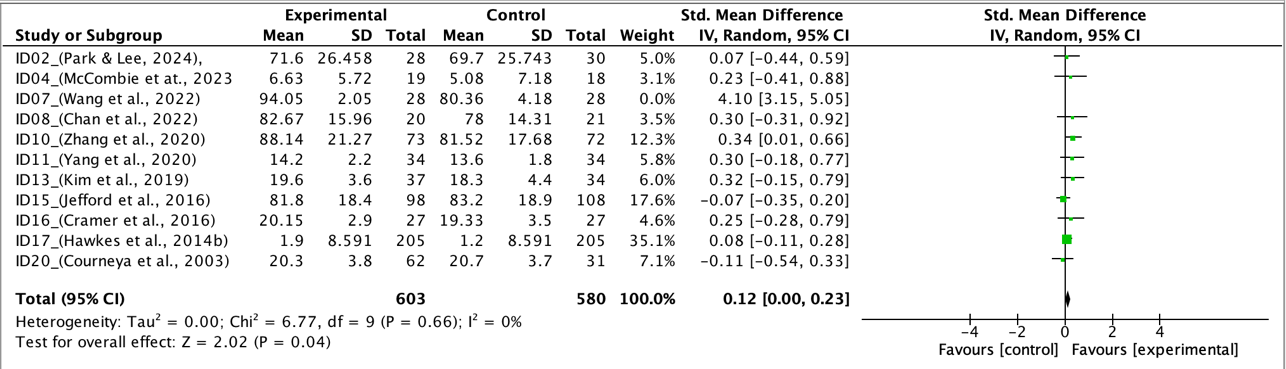** |

***Legend:*** Forest plot of the survivorship programme. Squares represent the effect sizes of individual studies, while diamonds indicate the summarised effect sizes. The horizontal lines denote the upper and lower bounds of the 95% confidence intervals. P-values greater than 0.05 are considered to indicate statistical non-significance.

# **Supplementary File 5.** Forest plot of the survivorship programme comparing the survivorship programme (experimental) with the usual care (control) in secondary outcome.

1. **Distress**

| 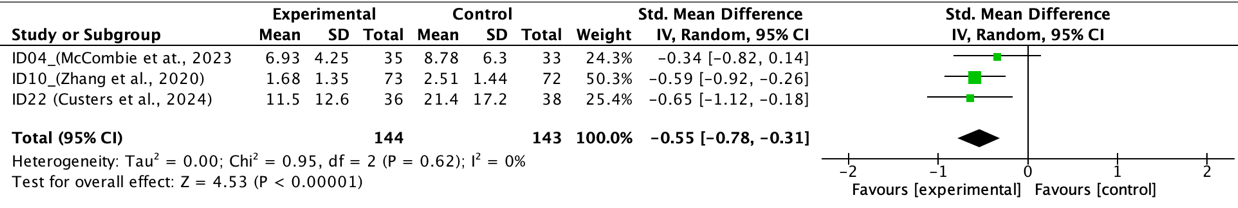 |
| --- |

1. **Anxiety**

| 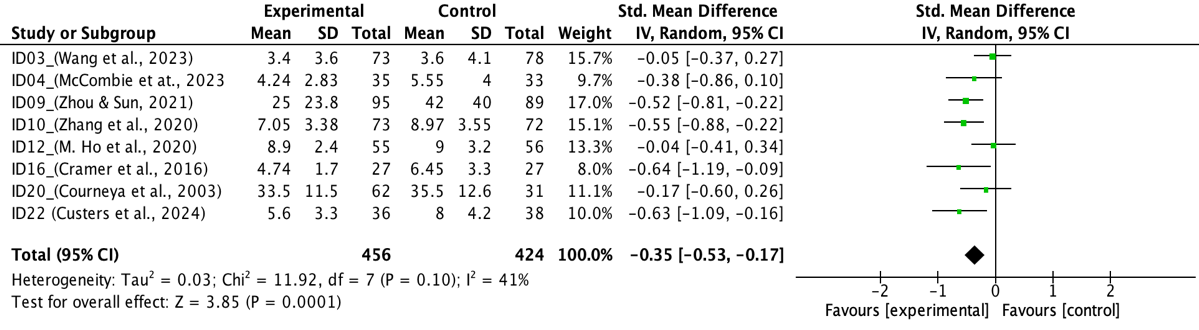 |
| --- |

1. **Depression**

| **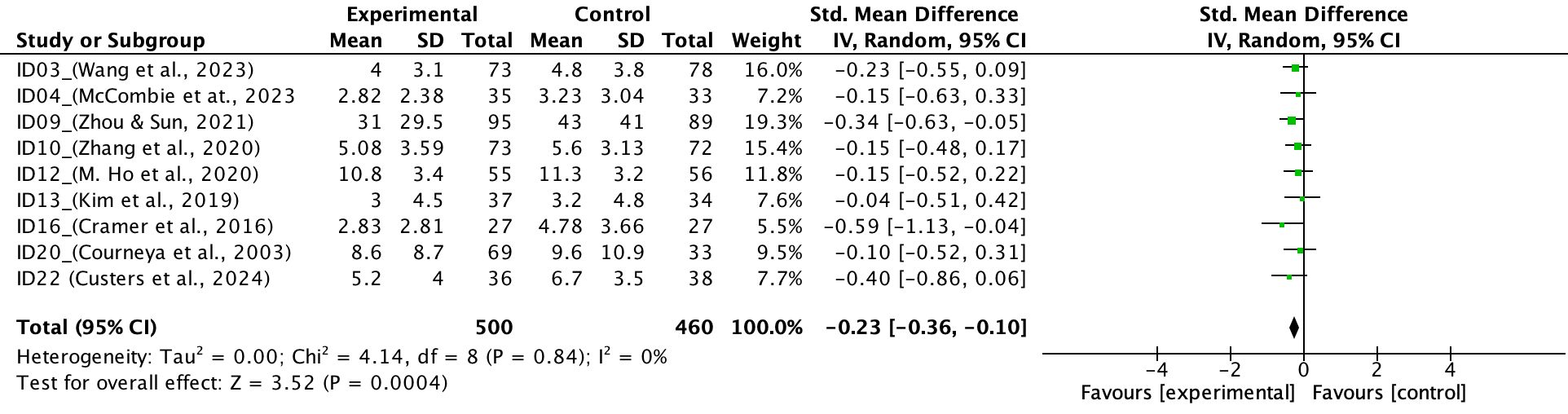** |
| --- |

1. **Fatigue**

| **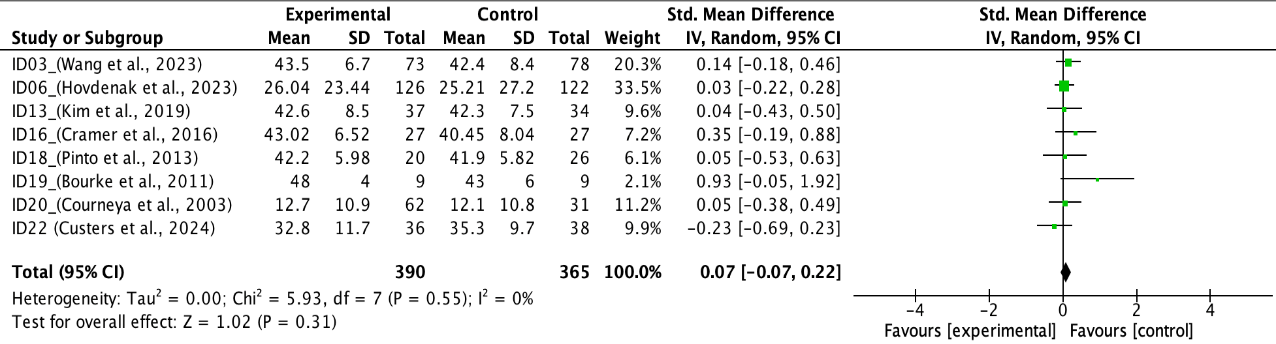** |
| --- |

**(E) Bowel Dysfunction**

| 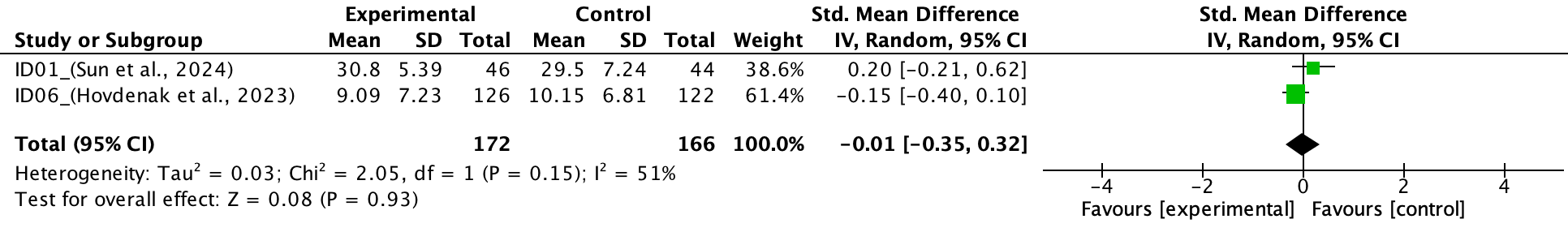 |
| --- |

***Legend:*** Forest plot of the survivorship programme. Squares represent the effect sizes of individual studies, while diamonds indicate the summarised effect sizes. The horizontal lines denote the upper and lower bounds of the 95% confidence intervals. P-values greater than 0.05 are considered to indicate statistical non-significance.
